# Characterization of the Trimeric TOM Complex by HS‐AFM Single‐Molecule Analysis

**DOI:** 10.1111/gtc.70154

**Published:** 2026-10-24

**Authors:** Nanako Kobayashi, Satoshi N. Omura, Kana Kuzasa, Kenichiro Imai, Shiho Kawai, Hirotatsu Imai, Romain Amyot, Kenichi Umeda, Osamu Nureki, Toshiya Endo, Noriyuki Kodera, Yuhei Araiso

**Affiliations:** ^1^ Department of Clinical Laboratory Science Graduate School of Medical Science, Kanazawa University Kanazawa Japan; ^2^ Department of Biological Sciences Graduate School of Science, the University of Tokyo Tokyo Japan; ^3^ Cellular and Molecular Biotechnology Reserach Institute (CMB), National Institute of Advanced Industrial Science and Technology (AIST) Tokyo Japan; ^4^ Integrated Reserach Center for Self‐Care Technology (IRC‐SRT), National Institute of Advanced Industrial Science and Technology (AIST) Tokyo Japan; ^5^ Department of Biochemistry Graduate School of Medicine, University of the Ryukyus Ginowan Japan; ^6^ WPI‐Nano Life Science Institute, Kanazawa University Kanazawa Japan; ^7^ Institute of Science Tokyo, Institute of Integrated Research, Cell Biology Center Yokohama Japan; ^8^ Faculty of Life Sciences Kyoto Sangyo University Kyoto Japan; ^9^ Institute for Protein Dynamics, Kyoto Sangyo University Kyoto Japan; ^10^ Environmental Stress Research Center (eSRC), Kanazawa University Ginowan Japan

**Keywords:** Cryo‐EM, HS‐AFM, membrane protein complex, mitochondria, protein translocation

## Abstract

The translocase of the outer mitochondrial membrane (TOM) complex is the entry gate for mitochondrial proteins synthesized in the cytosol. Previous biochemical studies indicated that the TOM complex functions as a trimer and exists in a dynamic equilibrium with a Tom22‐lacking dimer in intact mitochondria. In contrast, recent cryo‐electron microscopy (cryo‐EM) studies revealed near‐atomic structures of the Tom22‐containing core dimer. However, the relationship between the core dimer and the functional trimer remains unclear. Here, we analyzed the oligomeric organization and structural properties of purified yeast TOM complexes using high‐speed atomic force microscopy (HS‐AFM). Purified yeast TOM complexes predominantly adopted a trimeric organization but underwent deformation and dissociation during repeated HS‐AFM scanning, leaving dimeric and monomeric particles. Trimeric particles exhibited spherical shapes consistent with previous crosslinking models, whereas dissociated dimers closely resembled TOM core‐dimer structures determined by cryo‐EM. HS‐AFM also revealed the peripheral localization of the Tom20 receptor and enabled direct visualization of substrate–TOM interactions, with TOM association accompanied by reduced substrate mobility. Together, these findings provide quantitative and structural evidence for a predominantly trimeric organization of the purified TOM complex and suggest that the trimer contains a stable core‐dimer unit with a more loosely associated third Tom40‐containing unit.

## Graphical abstract

Purified yeast TOM complexes predominantly appeared as trimers but dynamically deformed and dissociated into core dimers and monomers during HS‐AFM observation, suggesting a loosely associated assembly of a core dimer and a monomer. HS‐AFM also revealed the spatial relationship of Tom20 with the TOM complex and directly visualized interactions between the TOM complex and substrate proteins.

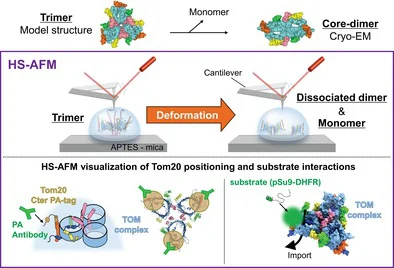

## 1 Introduction

Mitochondria are essential organelles that maintain cellular homeostasis and mediate diverse cellular functions. In humans, mitochondria contain approximately 1500 different proteins, whereas yeast mitochondria contain approximately 800 proteins. However, only a small number of mitochondrial proteins are encoded by the mitochondrial genome. More than 99% of mitochondrial proteins are synthesized in the cytosol and subsequently imported into mitochondria, where they are sorted into the outer membrane, intermembrane space, inner membrane, and matrix compartments (Endo and Yamano 2009; Neupert 2015; Pfanner et al. 2019; Endo and Wiedemann 2025).

The translocase of the outer mitochondrial membrane (TOM) complex serves as the major entry gate for the import of mitochondrial precursor proteins (preproteins). The TOM complex consists of the β‐barrel channel protein Tom40 and six receptor or auxiliary subunits, including Tom70, Tom22, Tom20, Tom7, Tom6, and Tom5, each of which contains a single transmembrane helix (Araiso et al. 2022). Although Tom40 was identified as the central import channel of the TOM complex around 1990 (Vestweber et al. 1989; Baker et al. 1990; Kiebler et al. 1990), the high‐resolution structure of the TOM complex remained unresolved for many years. Early electron microscopy studies using negatively stained particles demonstrated that purified TOM complexes from *Saccharomyces cerevisiae* and *Neurospora crassa* contained mixtures of trimeric and dimeric particles with three or two apparent protein‐conducting pores, respectively (Künkele et al. 1998; Model et al. 2002). Subsequently, moderate‐resolution cryo‐electron microscopy (~18 Å) suggested that the TOM complex can adopt a trimeric architecture and that the presence of Tom20 favors formation of the trimeric complex (Model et al. 2008). Systematic crosslinking analyses further supported a trimeric TOM complex containing three copies each of Tom40 and Tom22 (Shiota et al. 2015). Biochemical analyses also showed that the trimeric TOM complex dynamically exchanges with a Tom22 lacking dimer, which mediates the import of the intermembrane space‐targeted proteins (Sakaue et al. 2019).

More recently, near‐atomic‐resolution structures of the yeast TOM complex were determined by single‐particle cryo‐electron microscopy (Bausewein et al. 2017; Araiso et al. 2019; Tucker and Park 2019). Unexpectedly, these structures revealed a core dimer composed of two Tom40 channels surrounded by Tom22, Tom7, Tom6, and Tom5, which did not fully reconcile with the trimeric and Tom22‐lacking dimeric forms suggested by previous biochemical studies (Shiota et al. 2015; Sakaue et al. 2019). Soon afterward, cryo‐EM structures of the human TOM complex demonstrated a similar overall architecture (Wang et al. 2020; Guan et al. 2021). Structural analyses of holo‐TOM complexes have also progressively clarified the positions of Tom20 subunits that were previously unresolved; however, these structures likewise adopted dimeric organizations (Su et al. 2022; Su et al. 2024; Agip et al. 2025; Callegari et al. 2025).

These observations raise an important question regarding the relationship between the trimeric TOM complexes observed in intact mitochondria and the Tom22‐containing core‐dimer structures resolved by cryo‐EM analysis. Earlier electron microscopy studies suggested that purified TOM complexes contain mixtures of trimeric and dimeric forms (Künkele et al. 1998; Model et al. 2002; Model et al. 2008). In addition, previous high‐speed atomic force microscopy (HS‐AFM) studies suggested that purified TOM complexes can adopt trimeric structures that tend to dissociate into dimeric and monomeric forms (Araiso et al. 2019). However, the detailed molecular architecture and structural properties of these particles have not been fully characterized, and the relationship between the TOM complexes observed by cryo‐EM and HS‐AFM remains unclear.

In this study, we demonstrated that purified yeast TOM complexes predominantly adopt a trimeric organization and characterize their deformation and dissociation during repeated HS‐AFM scanning. The trimeric particles observed by HS‐AFM exhibited spherical molecular shapes consistent with a trimeric structural model proposed from previous crosslinking analyses (Shiota et al. 2015). In contrast, the dissociated dimeric particles closely matched the dimensions of the TOM core‐dimer structures previously determined by cryo‐EM (Araiso et al. 2019). Furthermore, antibody‐labeling experiments targeting Tom20 provided information on the organization of Tom20 within individual trimeric TOM complexes. We also directly visualized interactions between the TOM complex and a model substrate by HS‐AFM.

## 2 Results

### 2.1 The HS‐AFM Imaging of the Trimeric TOM Complex

To advance the structural analysis of the TOM complex, we constructed a budding yeast strain overexpressing each TOM complex subunit, including Tom40‐Strep‐tag II, Tom22‐10 × His, Tom20, Tom7, Tom6, and Tom5. Both the Strep‐tag II and 10 × His tag were positioned on the intermembrane space (IMS) side of the complex. We first isolated mitochondria and affinity‐purified the TOM complex using the Strep‐tag II under detergent micelle conditions. Gel filtration chromatography demonstrated that the purified TOM complex eluted as a sharp peak at 14–15 mL (Figure 1A). Immunoblot analyses following SDS‐PAGE and blue‐native PAGE (BN‐PAGE) further showed that the purified TOM complex formed a homogeneous assembly containing Tom40, Tom22, Tom20, Tom7, and Tom5 (Figures 1B and S1A). Owing to the lack of a suitable antibody, the presence of Tom6 could not be directly confirmed. The absence of Tom70 is consistent with its loose association with the TOM core complex reported previously (Model et al. 2002; Dekker et al. 1998). The estimated molecular mass of the TOM complex was approximately 530 kDa by gel filtration chromatography using molecular‐mass standards. In BN‐PAGE, the purified TOM complex migrated at a position corresponding to the mature TOM complex which was previously referred to as the 400 K complex (Sakaue et al. 2019). Together, the sharp gel‐filtration peak and the major band observed by BN‐PAGE indicated that the purified TOM complex was maintained as a relatively homogeneous assembly.

**FIGURE 1:**
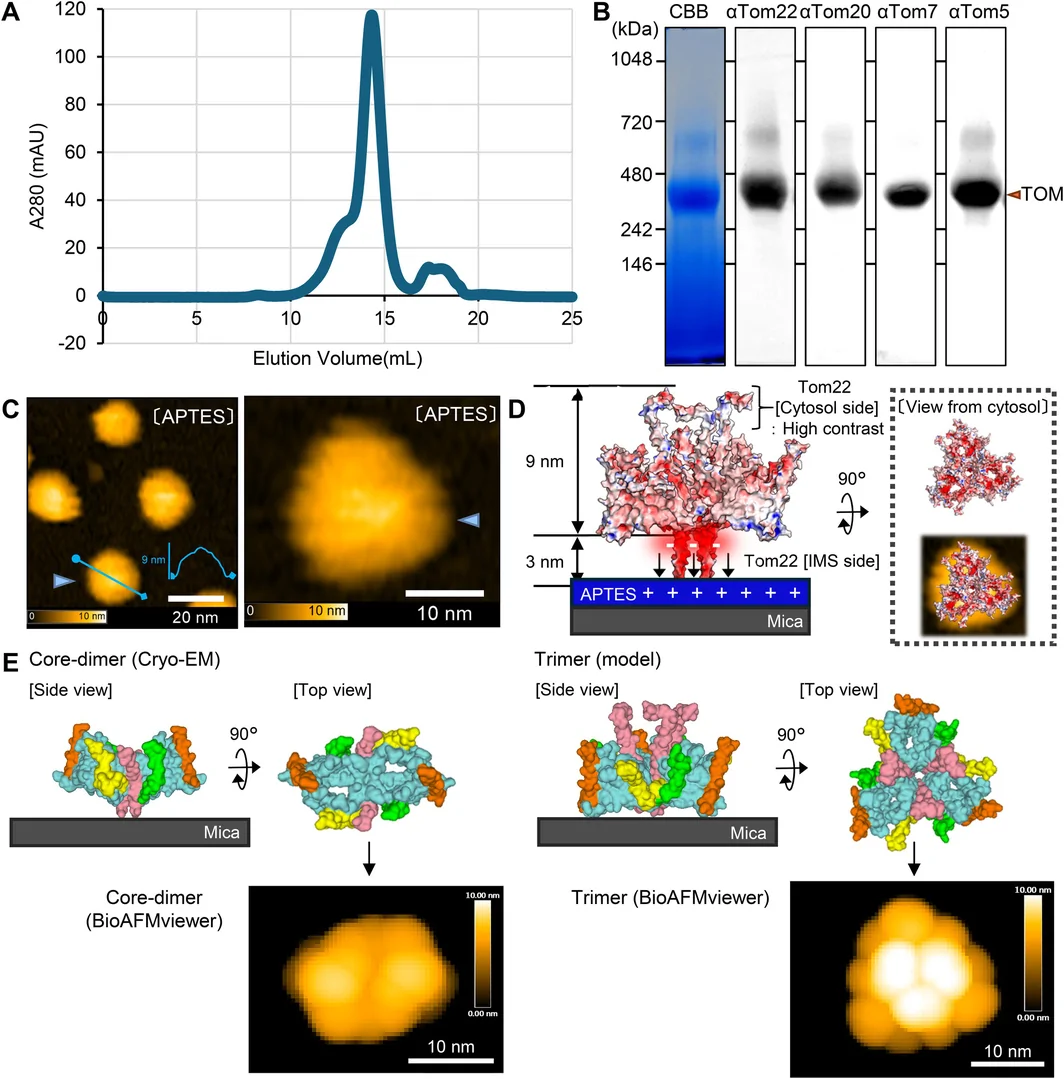
HS‐AFM analysis of the purified TOM complex. (A) The size‐exclusion chromatogram of the TOM complex under 0.01% GDN condition. (B) The largest peak of the size‐exclusion chromatogram was analyzed by Blue Native‐PAGE with CBB staining and western blot analysis. The purified TOM complex forms an approximately 400 kDa protein complex containing Tom5, Tom7, Tom20 and Tom22. (C) HS‐AFM images of the TOM complex on the APTES‐mica surface. The wide range view was on the left and the close‐up view was on the right. The blue arrow points to the same particle. Imaging conditions were either 2 frames per second with a scan size of 150 × 150 nm^2^ and a pixel resolution of 180 × 90 on the left panel and 20 frames per second with a scan size of 50 × 35 nm^2^ and a pixel resolution of 80 × 28 on the right panel. The molecular images were cropped from the original data. The uncropped images were included in Figure S6. (D) The schematic diagram using a trimeric structure model of the TOM complex. The negative‐charged patch of Tom22 on the IMS side is interacted with the positive‐charged patch of the APTES‐mica plate, and thus the cytosol side of the TOM complex was observed by HS‐AFM. (E) The simulated HS‐AFM images by BioAFMviewer. The simulated images were generated from the Cryo‐EM structure of the core‐dimer (left) and the model structure of the trimer (right).

Given the discrepancy between the trimeric organization suggested by previous biochemical crosslinking studies and the core‐dimer architecture predominantly observed by cryo‐EM (Shiota et al. 2015; Araiso et al. 2019), we sought to directly assess the oligomeric organization of the purified TOM complex. For this purpose, we employed HS‐AFM, which enables direct visualization of individual protein complexes and assessment of their stoichiometry under near‐native conditions. We first analyzed the oligomeric state of individual TOM complexes by HS‐AFM. HS‐AFM imaging revealed that the purified TOM complexes predominantly exhibited homogeneous circular particles, consistent with a trimeric rather than a dimeric organization. (Figure 1C). In the previously proposed trimeric model, three Tom40 channels are connected through Tom22 helices, resulting in clustering of the three Tom22 helices at the center of the complex (Shiota et al. 2015). Tom22 is an important subunit which consists of an N‐terminal cytosolic domain (~100 amino acids), a transmembrane helix, and a short C‐terminal IMS region (~30 amino acids) (Araiso et al. 2020; Shiota et al. 2011). Because the mica surface was coated with 3‐aminopropyltriethoxysilane (APTES), which imparts a positive charge and facilitates immobilization of TOM particles, the negatively charged IMS region of Tom22 and the negatively charged IMS‐facing surface of Tom40 were expected to interact with the mica surface (Araiso et al. 2022; Araiso et al. 2019; Tucker and Park 2019) (Figure 1D). Accordingly, the N‐terminal cytosolic domain of Tom22 would be oriented toward the solvent and likely correspond to the bright central density observed in the HS‐AFM images.

Based on the trimer structural model, the combined height of the transmembrane region and cytosolic domain is approximately 9 nm, whereas the IMS region of Tom22 extends ~3 nm from the membrane (Figure 1D). The TOM particles exhibited heights of approximately 8–10 nm by HS‐AFM analysis (Figure 1C), closely matching the expected dimensions of the transmembrane and cytosolic regions. These observations are consistent with a model in which the IMS helices of Tom22 are partially bent on the mica surface during HS‐AFM imaging (Figure 1D).

We next generated simulated AFM images of both the TOM core dimer and trimer based on the cryo‐EM structure and the trimeric model, respectively (Figure 1E). The experimentally observed TOM particles displayed circular shapes with a bright central region, rather than the oval‐shaped features predicted for the TOM core dimer. Frame averaging of sequential HS‐AFM images further emphasized this rounded morphology with a prominent central density, without resolving three individual lobes (Figure S1B). Notably, the simulated AFM image of the trimeric model also exhibited a rounded morphology with a prominent central density rather than three clearly separated lobes, consistent with the limited visibility of the three‐lobed architecture in the experimental HS‐AFM images. These observations suggested that the particles observed by HS‐AFM more closely resembled a trimeric TOM complex (Figure 1E).

### 2.2 Deformation and Dissociation of the Trimeric TOM Complex During HS‐AFM Imaging

During repeated HS‐AFM scanning, the purified trimeric TOM complex gradually deformed and dissociated. Because HS‐AFM measures molecular height through repeated tapping with a nanometer‐scale cantilever, continuous scanning gradually induced deformation of the TOM complex during imaging (Figures 2A,C and S2A,B, Movies [Link], [Link]). Such deformation and dissociation were observed under various experimental conditions, including observations of the TOM complex alone (Figures 2A and S2A, Movies 1 and 3) and in the presence of substrate proteins (Figure 2C and Movie 2) or antibodies against the TOM complex (Figure S2B and Movie 4). During repeated scanning, we observed that individual circular TOM particles progressively deformed into triangular‐shaped structures, revealing three distinguishable lobes that likely corresponded to three Tom40 channels. Notably, these triangular intermediates resembled particles previously reported in early electron microscopy studies (Künkele et al. 1998; Model et al. 2002).

**FIGURE 2:**
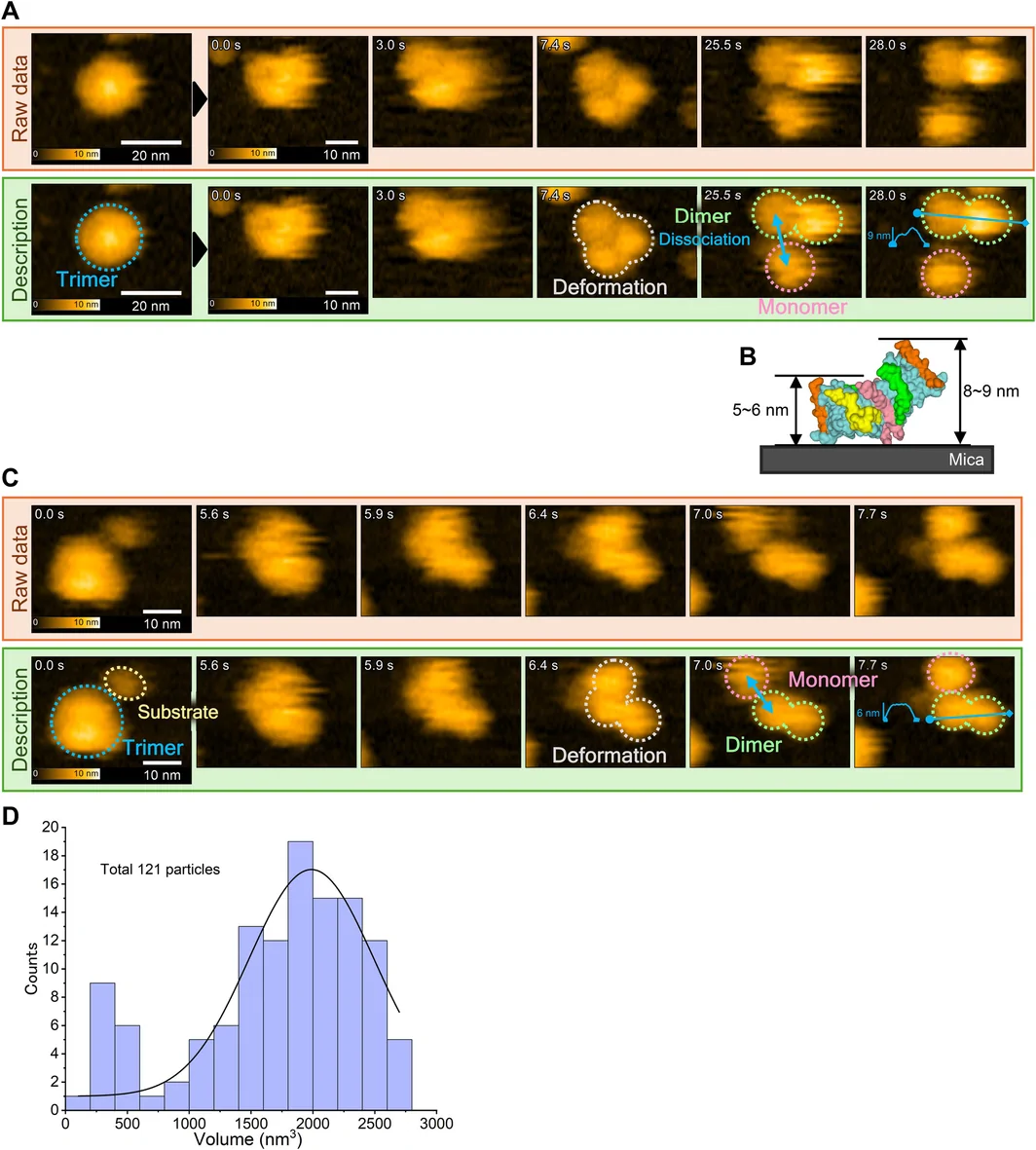
Deformation and dissociation of the TOM complex trimer during HS‐AFM imaging. (A, C) Representative HS‐AFM observations of two independent TOM complex particles are shown. For each observation, raw images and corresponding annotated images are presented to facilitate interpretation of the structural changes. During repeated scanning, initially rounded trimeric particles progressively deformed, revealing three distinguishable lobes, followed by separation into a pair of connected lobes and a single lobe, interpreted as dimeric and monomeric TOM complexes, respectively. The trimers, deformation intermediates, dimers, monomers and substrates are circled in blue, light gray, green, pink and yellow, respectively. (B) The schematic illustrates a possible tilted orientation of the Tom40 channels relative to the mica surface, which may account for the different apparent heights of individual lobes observed during deformation. In (C), the TOM complex imaged in the presence of substrate protein is shown. Imaging conditions were either 7.68 frames per second with a scan size of 75 × 52 nm^2^ and a pixel resolution of 120 × 42 in (A), or 6.67 frames per second with a scan size of 75 × 52 nm^2^ and a pixel resolution of 140 × 49 in (C). The molecular images used in this figure were also cropped from the original data. The uncropped images were included in Figure S7. (D) Distribution of the apparent volumes of TOM complex particles measured by HS‐AFM. Apparent volumes were calculated for 121 particles collected from multiple scan areas under the same imaging condition. The histogram was constructed using a bin width of 200 nm^3^ and fitted with a Gaussian function. The center of the fitted distribution was approximately 1988 nm^3^.

Subsequently, the trimeric TOM particles dissociated into a single isolated lobe and a pair of connected lobes, which likely corresponded to monomeric and dimeric TOM complexes, respectively (Figures 2A,C and S2A,B, and Movies [Link], [Link]). In some dimer particles, the individual lobes exhibited different apparent heights, possibly reflecting a tilted arrangement of the Tom40 channels relative to the mica surface (Figure 2A,B). Because the dissociated particles were presumably re‐surrounded by detergent micelles, reassociation of the monomeric and dimeric particles was rarely observed under the present experimental conditions. In addition, TOM trimers appeared to undergo deformation and dissociation more frequently in the presence of substrate proteins or antibodies against the TOM complex, possibly reflecting increased mechanical perturbation during HS‐AFM imaging (Figures 2C and S2B, Movies 2 and 4).

We next quantitatively characterized the population of TOM particles observed by HS‐AFM. A total of 121 particles collected from multiple scan areas were analyzed, and representative HS‐AFM images from these measurements are shown in Figure S2C. For each particle, the height and the full width at half maximum (FWHM) were determined from line profiles along the long and short axes. The mean height obtained from the two profiles was used as the particle height, and an apparent particle volume was calculated by approximating each particle as an ellipsoid. Histograms of these parameters revealed a single major population with dimensions consistent with those of the TOM particles assigned as trimers in Figure 1 (the apparent volumes of 10 representative particles were approximately 1100–1700 nm^3^ under the same imaging conditions as in Figure 1), whereas distinct populations corresponding to dimeric or monomeric TOM complexes were not detected (Figures 2 and S2C). Thus, although dimeric and monomeric particles emerged during deformation and dissociation under repeated HS‐AFM scanning, they did not constitute distinct populations in the quantitative analysis of the purified TOM sample. These results indicate that the purified TOM complex predominantly adopts a trimeric organization before deformation and dissociation during repeated HS‐AFM scanning.

In contrast, cryo‐EM analysis of the same preparation preferentially resolved the TOM core‐dimer rather than the trimeric assembly (Figure S3). Given that trimeric TOM particles were observed to dissociate into smaller assemblies during repeated HS‐AFM scanning, the preferential detection of the core‐dimer by cryo‐EM may reflect dissociation of the more labile trimeric assembly during sample preparation or imaging. However, the precise basis for the different oligomeric states observed by HS‐AFM and cryo‐EM remains unclear.

### 2.3 Biochemical Identification of TOM Complexes Containing Three Differently Tagged Tom40 Molecules

Because HS‐AFM and cryo‐EM analyses of the same TOM preparation yielded different oligomeric states, we next revisited the oligomeric organization of the purified TOM complex using an independent biochemical approach. Specifically, we sought to determine whether TOM complexes containing at least three copies of Tom40 were present in the purified sample. To this end, we constructed a yeast strain simultaneously expressing three differently tagged Tom40 proteins fused to Strep‐tag II, 3 × FLAG, or PA tags, respectively. (Figure 3A) In this strain, Strep‐tag II–, 3 × FLAG‐, and PA‐tagged Tom40 proteins were coexpressed on the mitochondrial outer membrane (Figure S4A). Because the three differently tagged Tom40 proteins could be incorporated into TOM complexes in various combinations, only a subset of the resulting complexes would be expected to contain all three tags simultaneously (“triple‐tagged TOM complexes”). Consequently, only a limited fraction of TOM complexes could be recovered through all three sequential affinity‐purification steps.

**FIGURE 3:**
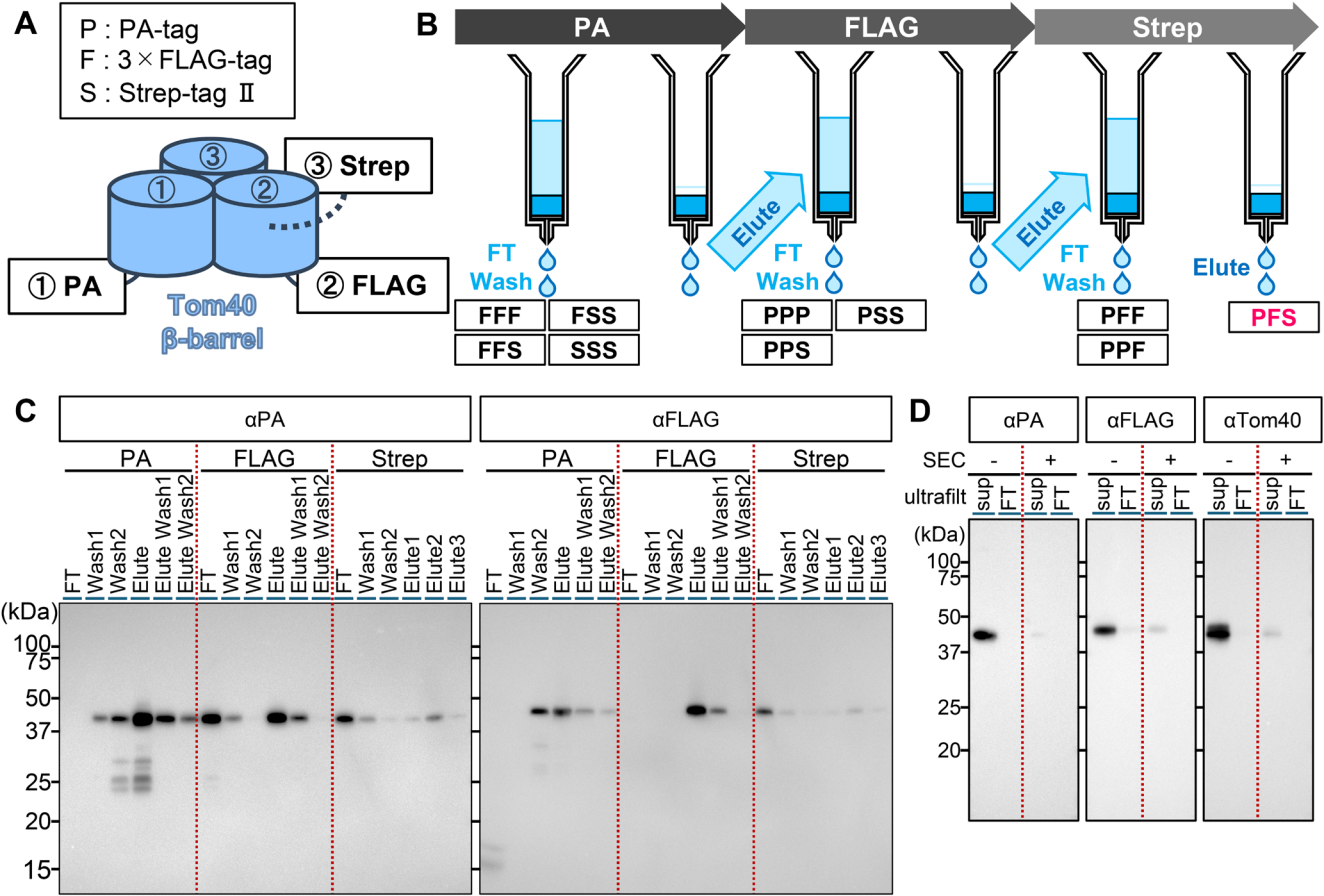
Identification of the trimeric TOM complex. (A) The schematic diagram of the triple‐tagged TOM complex and the procedure of affinity purifications employing the PA, the 3 × FLAG and the Strep II tags affinity resins. (B) The western blot analysis showing the three‐step affinity purification process, with anti‐PA and 3 × FLAG tags antibodies and the HRP conjugated Strep‐Tactin used for detection. (C, D) Gel filtration chromatogram of the triple tagged TOM complex and western blot analysis. Although the yield was low and no distinct peak could be detected, western blot analysis of the concentrated fractions around 14 mL, where the TOM complex was expected to elute, revealed a Tom40 band using anti‐PA, 3 × FLAG and Tom40 antibodies. Even though Tom40‐Strep was not observed, likely because its concentration was below the detection limit of Strep‐Tactin, it is believed that the purified TOM complex contains Tom40‐Strep, as Strep II tag affinity purification was performed at the end of the three‐step purification procedure. The uncropped images were included in Figure S8.

Using isolated mitochondria, we sequentially purified TOM complexes through PA‐, FLAG‐, and Strep‐tag affinity purification steps and analyzed the purified fractions by immunoblotting using anti‐PA and anti‐FLAG antibodies (Figures 3B,C and S4B). Although the amount of purified material was below the detection limit of gel filtration chromatography, immunoblot analyses clearly detected PA‐ and 3 × FLAG‐tagged Tom40 proteins at the elution position corresponding to the TOM complex trimer (14–15 mL) (Figures 3D and S4C). Strep‐tagged Tom40 was also examined by immunoblotting but was below the detection limit in the final SEC fraction. Nevertheless, recovery of the complex through the final Strep‐affinity purification step supports the presence of Strep‐tagged Tom40 in the affinity‐purified complex. These results therefore support the presence of TOM complexes containing at least three differently tagged Tom40 molecules. Although this experiment alone does not establish an exact trimeric stoichiometry, it provides complementary biochemical evidence for the trimeric organization observed by HS‐AFM.

### 2.4 Structural Characterization of the Trimeric TOM Complex by Antibody Labeling

Previous electron microscopy studies suggested that the receptor subunit Tom20 is localized at the outer peripheral region of the trimeric TOM complex and functions in precursor recognition (Model et al. 2008). We therefore attempted to visualize the position of Tom20 in HS‐AFM images. To this end, we purified a PA‐tagged TOM complex in which a PA tag was fused to the C‐terminal cytosolic region of Tom20 and incubated the purified complex with anti‐PA tag antibodies.

As described above, trimeric TOM complexes underwent deformation and dissociation during repeated HS‐AFM scanning. To stabilize the trimeric particles and facilitate visualization of the spatial organization of Tom20 by antibody labeling, we employed the GraFix method, which combines glutaraldehyde cross‐linking with sucrose density gradient centrifugation (Kastner et al. 2007) (Figure S5B,D).

Prior to the antibody‐binding experiments, we evaluated the efficiency of GraFix‐mediated cross‐linking. SDS‐PAGE analysis revealed broad high‐molecular‐weight signals, indicating that TOM complex subunits were cross‐linked by glutaraldehyde (Figure S5A,B). BN‐PAGE analysis showed that GraFix‐treated untagged TOM complexes migrated at a position similar to that of the untreated complexes, with no prominent higher‐molecular‐weight species indicative of intermolecular aggregation (Figure S5C). The GraFix‐treated PA‐tagged TOM complex used for the antibody‐labeling experiments migrated at a slightly higher molecular‐weight position, around 480 kDa (Figure S5D). This slight difference may reflect the effects of PA tagging and/or glutaraldehyde cross‐linking on the migration behavior of the complex. No prominent higher‐molecular‐weight species indicative of larger aggregates were observed.

When the cross‐linked TOM complex was incubated with anti‐PA tag antibodies and analyzed by HS‐AFM (Figure 4A), we observed one to three antibody particles associated with individual TOM complexes (Figure 4B,C). Notably, in some cases, three antibody‐derived particles were simultaneously associated with a single TOM complex. These observations provide direct information on the organization of Tom20 within individual trimeric TOM complexes and suggest that multiple Tom20 molecules, potentially up to three, can be associated with a single trimeric assembly. To exclude the possibility that the observed antibody particles simply reflected nonspecific adsorption to the mica surface, we performed a control experiment with the anti‐PA antibody alone under the same immobilization conditions. No antibody particles were detected on the APTES‐coated mica surface in the absence of the TOM complex, indicating that nonspecific adsorption of the antibody to the mica surface was negligible under these conditions.

**FIGURE 4:**
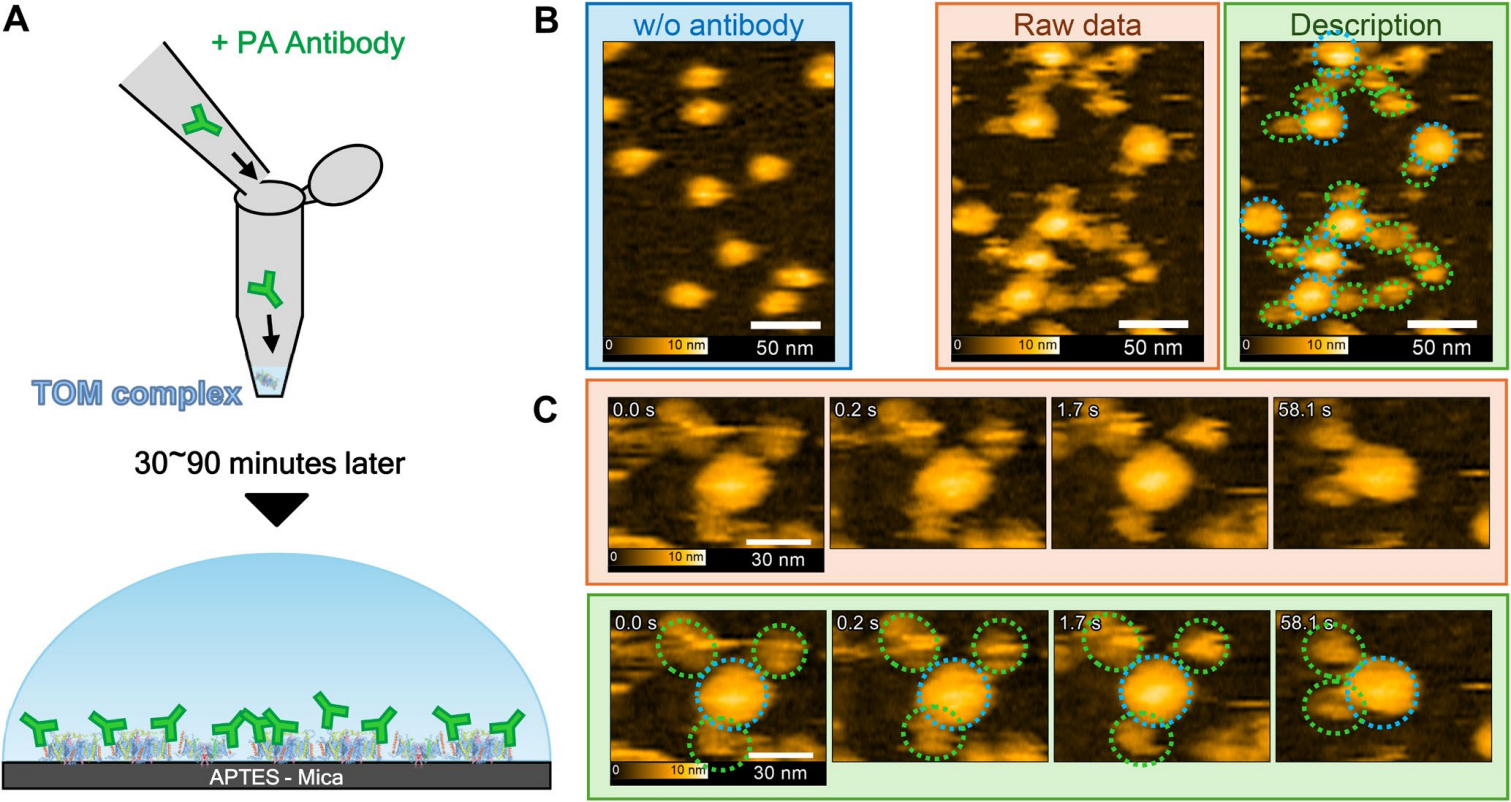
Real‐time observation of the position of the Tom20 receptor subunit and its substrate recognition. (A) Schematic diagram of the anti‐PA tag antibody addition experiment. (B) The representative images of the crosslinked‐TOM complex were shown separately in the absence and presence of antibodies. (C) The sequential images of the cross‐linked TOM complex with anti‐PA tag antibodies were shown. The upper panel indicated only the raw data, while the bottom panel included descriptions of each individual particle. The images in the upper and bottom panels are identical. The anti‐PA antibodies and the TOM complexes are surrounded by green and blue circles, respectively. Imaging conditions were either 1.05 frames per second with a scan size of 300 × 210 nm^2^ and a pixel resolution of 400 × 70 in (B), or 5.01 frames per second with a scan size of 100 × 70 nm^2^ and a pixel resolution of 200 × 35 in (C). The molecular images used in this figure were also cropped from the original data. The uncropped images were included in Figure S9.

Because the GraFix method successfully stabilized TOM complex trimers for HS‐AFM observation, we next attempted structural analysis by single‐particle cryo‐EM (Figure S5A,C). Although this analysis again yielded predominantly a dimeric structure resembling those reported previously (Araiso et al. 2019; Tucker and Park 2019), the 2D class averages also contained a putative trimeric form corresponding to the three Tom40 pores (Figure S5E). Despite the presence of this trimeric species, we were unable to obtain a high‐resolution 3D reconstruction of the trimeric complex. These observations suggest that trimeric TOM complexes, although likely present on the cryo‐EM grids, may not adopt a sufficiently stable or uniform conformation within detergent micelles suitable for high‐resolution single‐particle reconstruction, such that their structural heterogeneity or conformational flexibility limited efficient alignment and high‐resolution reconstruction during image processing. In this regard, our observations are consistent with previous negative‐staining electron microscopy studies that identified trimeric TOM particles at the single‐particle level (Künkele et al. 1998; Model et al. 2002).

### 2.5 Visualization of Substrate Association With the TOM Complex by HS‐AFM

Having characterized the oligomeric organization of the TOM complex, we next asked whether interactions between the TOM complex and a substrate protein could be directly visualized by HS‐AFM. To this end, we added dihydrofolate reductase (DHFR) fused to the presequence of *Neurospora crassa* ATP synthase subunit 9 (pSu9‐DHFR) during HS‐AFM observation of the TOM complex (Shiota et al. 2015; Kanamori et al. 1999) (Figure 5A). Although pSu9‐DHFR particles rapidly diffused across the mica surface, transient associations with TOM complex particles were repeatedly observed (Figure 5B,C and Movies 5 and 6). TOM association was determined based on the overlap of the FWHM of the substrate and TOM particle profiles, as illustrated in Figure S5F and described in detail in the Methods. To quantitatively assess changes in substrate movement upon TOM association, we compared the frame‐to‐frame movement speeds of substrate particles between TOM‐associated and unassociated states (Figure 5D). Substrate movement speed was significantly lower in the TOM‐associated state than in the unassociated state (28.1 ± 23.7 vs. 44.1 ± 29.3 nm/s, respectively; *p* = 0.0011, Welch's two‐tailed *t*‐test). These observations indicate that association with the TOM complex is accompanied by reduced substrate mobility.

**FIGURE 5:**
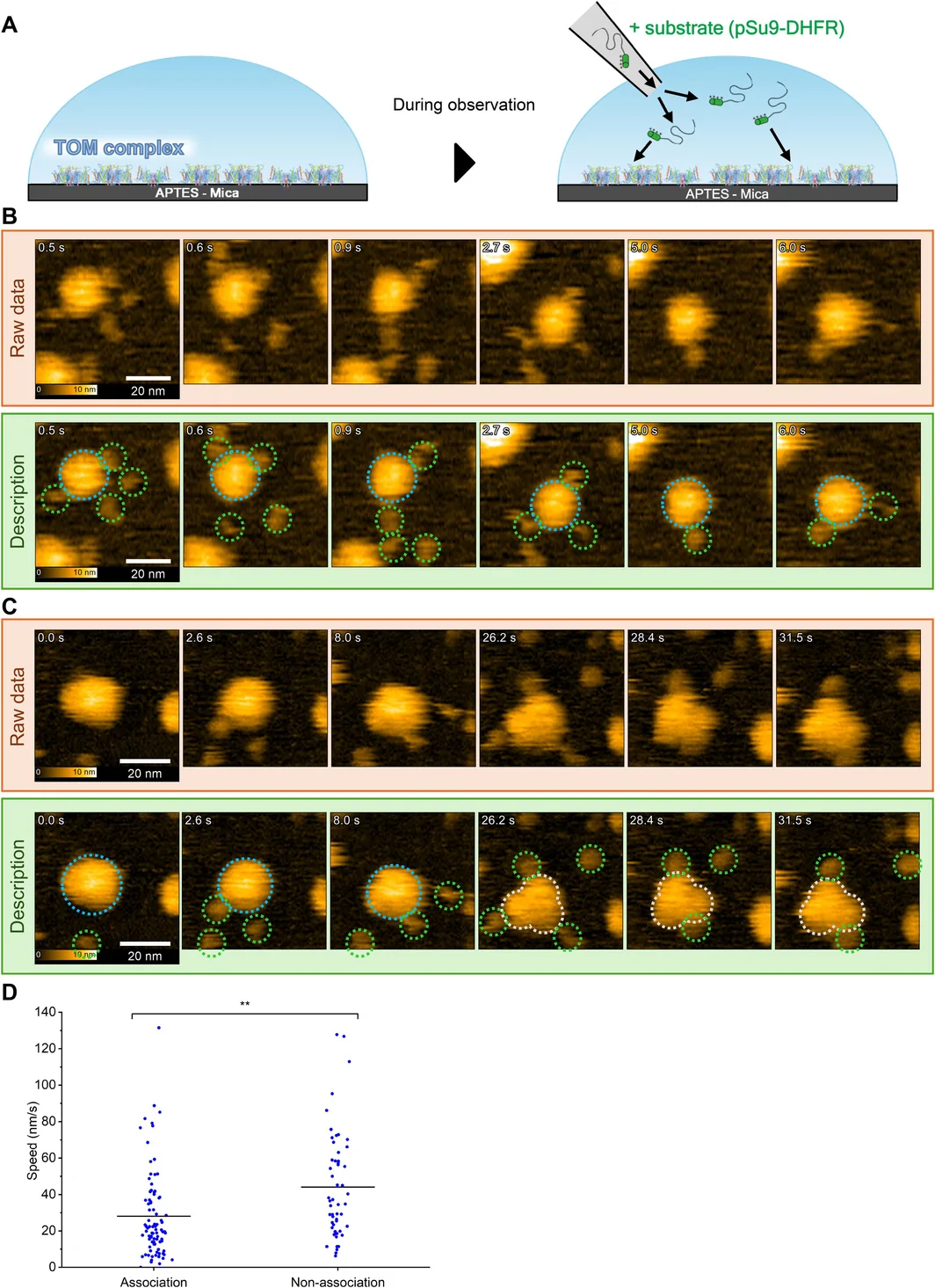
Real‐time visualization of transient substrate association with the TOM complex. (A) Schematic diagram of the substrate addition experiment was briefly described above. (B, C) The sequential images of the TOM complex and pSu9‐DHFR were shown. The upper panel indicated only the raw data, while the bottom panel included descriptions of each individual particle. The images in the upper and bottom panels are identical. The anti‐PA antibodies interacted with the TOM complex, which suggests the spatial positions of the Tom20 subunits of the TOM complex in the HS‐AFM images. The substrate protein pSu9‐DHFR, the TOM complexes and deformation intermediates are surrounded by green, blue and light gray circles, respectively. Imaging conditions were either 6.68 frames per second with a scan size of 80 × 80 nm^2^ and a pixel resolution of 120 × 60 in (B), or 8.36 frames per second with a scan size of 60 × 60 nm^2^ and a pixel resolution of 160 × 80 in (C). The molecular images used in this figure were also cropped from the original data. The uncropped images were included in Figure S10. (D) Box plots of frame‐to‐frame movement speeds of pSu9‐DHFR particles in TOM‐associated and unassociated states. Movement speed was calculated from the displacement of the substrate particle between consecutive frames (0.12 s per frame). Each movement was classified according to the substrate–TOM association state in the first frame. The distributions include 87 TOM‐associated and 54 unassociated frame‐to‐frame measurements obtained from a total of nine substrate particles.

## 3 Discussion

Since the core‐dimer structure of the TOM complex from *Neurospora crassa* was determined at 6.8 Å resolution in 2017 (Bausewein et al. 2017), high‐resolution TOM complex structures at near‐atomic resolution have been reported by several groups (Araiso et al. 2019; Tucker and Park 2019; Wang et al. 2020; Guan et al. 2021; Su et al. 2022; Su et al. 2024; Agip et al. 2025; Callegari et al. 2025). Nevertheless, cryo‐EM structural analyses have predominantly captured the TOM complex in its core‐dimer form, whereas tetrameric and hexameric assemblies have occasionally been observed (Tucker and Park 2019; Guan et al. 2021). Notably, the tetrameric structure essentially represents a dimerization of two TOM core dimers. In contrast, the precise structure corresponding to the trimeric TOM complex proposed in earlier studies has not yet been determined, despite previous electron microscopy studies suggesting the presence of trimeric TOM particles (Künkele et al. 1998; Model et al. 2002; Model et al. 2008; Shiota et al. 2015). Previously, we also observed trimeric TOM particles by HS‐AFM during cryo‐EM structural studies but did not perform detailed characterization (Araiso et al. 2019). In the present study, we systematically characterized the oligomeric organization of the purified TOM complex by HS‐AFM and further examined the structural features of the trimeric particles and their changes during repeated HS‐AFM imaging.

Based on the present observations, we propose a possible relationship between the trimeric TOM complex and the TOM core dimer. As shown in Figure 2, trimeric TOM particles were observed to dissociate into dimeric and monomeric particles during HS‐AFM observation. The dimeric particles observed by HS‐AFM likely correspond to the TOM core‐dimer structures previously identified by cryo‐EM analysis (Figure 2). This interpretation is further supported by the similarity between the experimentally observed HS‐AFM images and the simulated ones generated from the reported TOM core‐dimer structures (Figures 1E and 2). During cryo‐EM grid preparation and image processing, one Tom40 molecule within the trimeric assembly may become structurally unstable or partially dissociated, thereby favoring formation of the TOM core dimer (Figure S3).

The dissociation observed during HS‐AFM imaging should be distinguished from the physiological transition between TOM oligomeric states previously reported in intact mitochondria. The minor dimeric TOM species identified in previous biochemical studies lack Tom22, whereas the dimeric particles remaining after dissociation in our HS‐AFM observations resemble the Tom22‐containing TOM core dimer resolved by cryo‐EM (Shiota et al. 2015; Araiso et al. 2019). Thus, the present HS‐AFM observations are interpreted primarily in terms of the structural stability and organization of the purified trimeric assembly under repeated scanning, rather than as a direct visualization of the physiological trimer–dimer transition in the mitochondrial outer membrane.

However, this possibility does not imply that the TOM core dimer is an experimental artifact. Because previous biochemical and structural analyses based on the TOM core dimer successfully explained key aspects of mitochondrial protein translocation, the TOM core dimer likely represents a functional and energetically stable structural state on the mitochondrial outer membrane (Araiso et al. 2019). It is therefore conceivable that monomeric TOM particles are associated with the stable core‐dimer unit to form a trimeric assembly (Figure 2). In this model, the TOM trimer may adopt an asymmetric organization, in which a stable core dimer serves as the central structural unit and an additional monomer is more loosely associated with the complex, rather than three completely equivalent channels functioning in a fully symmetric manner. Although detailed structural analysis of the TOM trimer will require future high‐resolution studies, the current findings support the existence of trimeric TOM assemblies while suggesting that the energetically stable core‐dimer region is preferentially resolved by cryo‐EM analysis. We hope that the present study will facilitate future structural determination of the TOM trimeric complex.

Within the TOM complex, Tom70, Tom22, and Tom20 function as receptor subunits involved in substrate recognition (Brix et al. 1999; Yamano et al. 2008). Because Tom70 dissociated during solubilization of yeast mitochondria, its functional contribution could not be evaluated in the present HS‐AFM analyses (Bausewein et al. 2017). Tom22 additionally functions as a structural linker between Tom40 channels and is positioned at the center of the proposed trimeric TOM complex model (Shiota et al. 2015). Even in the TOM core‐dimer structures, two Tom22 helices are located between adjacent Tom40 channels, serving as a scaffold essential for maintaining TOM complex integrity (Araiso et al. 2019; Tucker and Park 2019).

In contrast, Tom20 is known to interact relatively weakly with the TOM complex and is thought to move with some degree of flexibility around the periphery of the complex (Shiota et al. 2015; Araiso et al. 2019). Although Tom20 is frequently disordered in TOM core‐dimer structures, several cryo‐EM studies have identified its approximate position by stabilizing the TOM complex through cross‐linking or substrate addition (Su et al. 2022; Su et al. 2024; Agip et al. 2025; Callegari et al. 2025). Owing to its structural flexibility and the variable positioning of its transmembrane helix, the precise binding site of Tom20 has remained difficult to define. Consequently, local structural resolution in this region is often limited, and atomic modeling of Tom20 is frequently incomplete. In the previously reported TOM core‐dimer structures, the receptor domains of Tom20 are positioned above the Tom40 channels (Su et al. 2024; Agip et al. 2025). By contrast, earlier moderate‐resolution electron microscopy and cross‐linking studies of the TOM trimer suggested that Tom20 localizes to the peripheral region of the triangular trimeric assembly (Model et al. 2008; Shiota et al. 2015).

HS‐AFM visualization of interactions between anti‐PA tag antibodies and peripheral regions of the PA‐tagged TOM complex supported the peripheral localization of Tom20 as described in the previous structural studies (Model et al. 2008; Shiota et al. 2015; Su et al. 2022; Su et al. 2024; Agip et al. 2025; Callegari et al. 2025). Furthermore, these antibody‐labeling experiments extend this structural view by suggesting that multiple Tom20 molecules, potentially up to three, can be associated with an individual trimeric TOM complex (Figure 4B,C). These observations suggest that formation of the trimeric complex may allow multiple presequence receptors to be positioned around a single TOM assembly.

In addition to characterizing the oligomeric organization of the TOM complex, HS‐AFM allowed us to directly visualize transient interactions between individual TOM complexes and the precursor model protein pSu9‐DHFR (Figure 5B,C and Movies 5 and 6). Quantitative analysis of substrate movement showed that pSu9‐DHFR particles exhibited lower movement speeds when associated with TOM complexes than when unassociated (Figure 5D). This reduced mobility is consistent with physical engagement of the precursor protein with the TOM complex and suggests that HS‐AFM can directly capture changes in substrate behavior accompanying its interaction with the translocase. The observed association and subsequent release of substrate particles in Movies 5 and 6 are also consistent with the transient nature expected for substrate interactions with a protein translocase. The DHFR domain of pSu9‐DHFR is folded, and interactions of pSu9‐DHFR with the mica surface may affect the observed substrate behavior.

Thus, our HS‐AFM analysis provides quantitative support for a predominantly trimeric organization of the purified TOM complex and suggests an asymmetric architecture comprising a stable core dimer and a more loosely associated third Tom40‐containing unit. Furthermore, antibody labeling suggests that multiple Tom20 molecules can be associated with an individual trimeric TOM complex, while time‐resolved HS‐AFM imaging enabled direct visualization of transient interactions between the TOM complex and a precursor protein. Together, these observations provide new structural and functional insights into the trimeric TOM complex, which has not yet been fully resolved by cryo‐EM analysis.

Whether the TOM complex physiologically requires trimeric architecture and what functional advantages are conferred by the presence of three channels remain important open questions. Nevertheless, the present study enabled characterization of the trimeric TOM complex in vitro and provides a foundation for future structural and functional analyses of TOM complex assembly and organization.

## 4 Experimental Procedures

### 4.1 Yeast Strains, Plasmids and Primers

To construct the *S. cerevisiae* strain for overexpression of the TOM complex, we introduced the *GAL1* promoter using the Yeast Tool Kit (YTK) and Golden Gate assembly, following a strategy similar to that used in a recent structural study (Tucker and Park 2019; Lee et al. 2015; Kobayashi et al. 2024) (Table S1). The coding sequences (CDSs) of Tom40, Tom22, Tom20, Tom7, and Tom5 were amplified by PCR, whereas the Tom6 gene was chemically synthesized. Each gene was individually inserted into the pYTK001 entry plasmid (Table S2). To enable affinity purification of the TOM complex, a Strep‐tagIIsequence (WSHPQFEK) with a GG linker was fused to the C‐terminus of Tom40. In addition, a 10 × His tag with a GG linker was fused to the C‐terminus of Tom22 to facilitate immobilization of the TOM complex on mica surfaces during HS‐AFM analysis. (see the HS‐AFM observation section below). The *GAL1* promoter (YTK030), the CDS of each TOM complex subunit, and the *ENO1* terminator (YTK061) were assembled into the pYTK095 cassette plasmid. The resulting cassette plasmids encoding individual TOM complex subunits were subsequently inserted into the pYTK096 plasmid containing the *URA3* auxotrophic marker (Table S3). The final vector was linearized with NotI and introduced into the W303 yeast strain using a standard lithium acetate transformation method. Transformants were selected on SCD‐ura medium containing 0.67% (w/v) yeast nitrogen base without amino acids, 0.5% (w/v) casamino acids, 2% (w/v) glucose, 0.002% (w/v) adenine sulfate, and 0.002% (w/v) L‐tryptophan.

To generate the strain expressing triple‐tagged Tom40 proteins, two additional Tom40 constructs fused to either a PA tag or a 3 × FLAG tag through GG linkers were subcloned into the pYTK095 vector. These tagged Tom40 constructs were tandemly integrated into a pYTK096‐based vector containing the LEU2 auxotrophic marker (Table S3). The TOM complex overexpression strain described above was subsequently transformed with this vector and cultured in SCD‐leu‐ura medium containing 0.67% (w/v) yeast nitrogen base without amino acids, 1.74% (w/v) SC‐leu‐ura supplement mixture (MP Biomedicals), and 2% (w/v) glucose. The resulting yeast strain simultaneously overexpressed three Tom40 variants fused to PA, 3 × FLAG, and Strep‐tag II sequences, respectively.

For HS‐AFM visualization of antibody binding, we additionally constructed another TOM complex overexpression strain in which a PA tag was fused to the C terminus of Tom20, together with the Strep‐tag II fused to Tom40 and the 10 × His tag fused to Tom22.

### 4.2 Protein Expression and Purification

Yeast strains were cultured in YPD (1% (w/v) yeast extract, 2% (w/v) polypeptone, 0.1% (w/v) glucose) medium until OD_600_ reached 0.6, then galactose was added to induce the transcription by the *GAL1* promoter, and cultured in 4 L YPGal (1% (w/v) yeast extract, 2% (w/v) polypeptone, 2% (w/v) galactose) medium at 30°C for 20 h. The following procedure for mitochondrial isolation was performed in accordance with the recent publication (Kobayashi et al. 2024). In brief, the yeast cells were collected and resuspended with Tris‐SO_4_ buffer (100 mM Tris SO_4_ [pH 9.4], 10 mM DTT). After incubating with gentle shaking at 90 rpm and 30°C for 10 min, the cell pellet was collected and incubated in sorbitol buffer (1.2 M sorbitol, 20 mM Tris–HCl [pH 7.5]) containing zymolyase 20T (zymolyase 5 mg/yeast 1 g) with shaking at 135 rpm and 30°C for 30 min. After washing out the zymolyase with sorbitol buffer, the cell pellet was disrupted in breaking buffer (600 mM sorbitol, 10 mM Tris–HCl [pH 7.5], 1 mM EDTA) using a tight‐fitting Dounce homogenizer. Then, the mitochondrial membrane fraction was isolated through multiple centrifugations and washed by SEM buffer (250 mM sucrose, 1 mM EDTA, 10 mM MOPS·NaOH [pH 7.2]).

Isolated mitochondria were solubilized by solubilization buffer (20 mM Tris–HCl [pH 7.5], 150 mM NaCl, 1% digitonin) at 4°C overnight, and centrifuged at 20,000 g at 4°C for 20 min. The supernatant was mixed with 1 mL of Strep‐Tactin 4Flow high capacity resin (IBA) and incubated at 4°C for more than 4 h. The resin was washed with Affinity buffer (50 mM Tris–HCl pH 7.5, 150 mM NaCl, 0.1% GDN), and the TOM complex was eluted by Affinity Buffer containing 3 mM D‐desthiobiotin.

The eluted TOM complex was concentrated using an Amicon Ultra (Merck Millipore, 100 kDa MW cut‐off) ultrafiltration kit by centrifugation at 5000 g (final volume was approximately 500 μL). The TOM complex was further purified by gel filtration chromatography using a Superose 6 Increase 10/300 GL column (cytiva) equilibrated with the SEC buffer (20 mM Tris [pH 7.5], 150 mM NaCl, 0.01% GDN).

Especially for Cryo‐EM analysis, the overexpression strain was cultured in 36–40 L of YPD medium. The purification procedures were almost same as those for HS‐AFM analysis, but with 4 mL of Strep‐Tactin 4Flow high capacity resin. To obtain high yield, even the TOM complex that passed through the column was recovered from flowthrough fraction.

### 4.3 Purification of the Triple‐Tagged TOM Complex

The yeast strain overexpressing the triple‐tagged TOM complex was cultured in 18 L of YPD medium. The mitochondrial membrane fraction was isolated and solubilized in the same way as described above. Then, the triple‐tagged TOM complex was sequentially purified using three affinity resins. First, the supernatant was mixed with 1 mL of Anti PA tag Antibody Beads (FUJIFILM) and incubated at 4°C for 2 h. The resin was washed with Affinity buffer and incubated in Affinity Buffer containing 0.1 mg/mL of PA tag Peptide (FUJIFILM) for 1 h. After that, the eluate TOM complex was collected. Furthermore, the resin was washed by 10 mL of Affinity buffer without any PA peptide (elute wash). The elution and elute wash fractions were mixed and subjected to 400 μL of ANTI‐FLAG M2 Affinity Gel (Merck) as a second affinity purification. After incubating at 4°C for 1 h, the resin was washed with Affinity buffer and incubated in Affinity Buffer containing 0.1 mg/mL of 3 × FLAG Peptide (ProteinArk) for 1 h. After that, the elution and elute wash fractions were collected and subjected to 1 mL of Strep‐Tactin 4Flow high capacity resin (IBA) as a third affinity purification. After incubating at 4°C overnight, the resin was washed with Affinity buffer, and the triple‐tagged TOM complex was eluted by Affinity Buffer containing 3 mM D‐desthiobiotin.

The eluted triple‐tagged TOM complex was concentrated using an Amicon Ultra (Merck Millipore, 100 kDa MW cut‐off) by centrifugation at 5000 g (final volume was approximately 500 μL). The triple‐tagged TOM complex was analyzed by gel filtration chromatography using a Superose 6 Increase 10/300 GL column (cytiva) equilibrated with the SEC buffer. The elution fractions around 14–15 mL were mixed and concentrated with Amicon Ultra (Merck Millipore, 100 kDa MW cut‐off) and analyzed by SDS‐PAGE.

### 4.4 Grafix

To prevent dissociation, we applied the gradient fixation method (GraFix) to the structural analysis (Kastner et al. 2007). First, the TOM complex was purified by Strep‐tag affinity purification using 50 mM HEPES‐KOH pH 7.4 instead of Tris–HCl buffer and gel filtration chromatography and concentrated by Amicon Ultra (100 kDa MW cut‐off) up to 400 μL volume. Then, a 17‐steps sucrose gradient was prepared manually, in which each fraction volume was 400 μL (Table S4). The low‐density buffer was composed of 20 mM HEPES‐KOH pH 7.4, 150 mM NaCl, 0.01% GDN, and 5% (w/v) sucrose, while the high‐density buffer contained 20 mM HEPES‐KOH pH 7.4, 150 mM NaCl, 0.01% GDN, 45% (w/v) sucrose, and 0.8% glutaraldehyde. 200 μL of the purified TOM complex was applied on the top. Centrifugation was carried out at 100,000 g (Accel:9, Decel:1) at 4°C for 13 h using a S50ST rotor (Himac).

Crosslinking efficiency was evaluated by SDS‐PAGE with silver staining. The fractions between 4 and 9 were collected and dialyzed using Tube‐O‐DIALYZER (G‐biosciences) in 200 mL of SEC buffer at 4°C. After 3 h of dialysis, the buffer was replaced, and dialysis was continued overnight. For cryo‐EM analysis, the dialyzed TOM complex was concentrated by Amicon Ultra (100 kDa MW cut‐off) up to 30 μL (Final concentration: 18.8 mg/mL).

### 4.5 Immunoblotting

The gel was transferred onto a Transblot Turbo Pack membrane (PVDF). Rabbit anti‐Tom40, Tom22, Tom20, Tom7, and Tom5 primary antibodies (1:1000–10,000) were used to evaluate the TOM complex formation in each purification. Rat anti‐PA and mouse anti‐FLAG antibodies (1:5000, 1:10,000, respectively) were used to evaluate the PA‐tagged TOM complex and the triple‐tagged TOM complex. Detection was carried out using Horseradish peroxidase (HRP)‐conjugated goat anti‐rabbit, rat, mouse antibodies (1:5000). HRP‐conjugated Strep‐Tactin (IBA) was also used to evaluate the Strep II tag that fused to Tom40 in all the TOM complex purified in this study. Signals were detected with a LuminoGraph I (ATTO) using chemiluminescence.

### 4.6 HS‐AFM Observation

The purified sample was collected only from the apex of the gel filtration chromatogram and diluted with an imaging buffer containing 10 mM Tris–HCl, pH 7.5, 30 mM NaCl, 10% glycerol and 0.01% GDN for dispersed observation. HS‐AFM analysis was performed in tapping mode at room temperature using a laboratory‐built HS‐AFM setup (Ando et al. 2001; Ando et al. 2008; Ando et al. 2024). We used short cantilevers (BL‐AC7DS‐A2, Olympus; spring constant, ~0.25 N/m; resonant frequency and quality factor in liquid, ~800 kHz and ~1.5, respectively). The probe tip was grown on the original tip end of a cantilever through electron beam deposition using ferrocene and was further sharpened using a radio‐frequency plasma etcher (Tergeo, PIE Scientific LLC) under an argon gas atmosphere (Direct mode, 10 sccm, and 20 W for 1.5 min). To increase spatial sampling, we employed non‐square pixels in all observations; the pixel size along the fast axis (X) was set to 2 ~ 4× smaller than that along the slow axis (Y) (Umeda et al. 2023). The general procedure for HS‐AFM observation of protein molecules was described previously (Uchihashi et al. 2012). In brief, a glass sample stage (diameter, 2 mm; height, 2 mm) with a thin mica disc (1.5 mm in diameter and 0.05 mm thick) glued to the top by epoxy was attached onto the top of the Z‐scanner by a drop of nail polish. The mica surface was treated by (3‐aminopropyl) triethoxysilane (APTES) diluted to 1:10,000 to facilitate immobilizing of the TOM complex particles onto the mica surface. Especially for the TOM complex crosslinked by GraFix method, APTES was diluted to 1:2000 due to higher mobility of the TOM complex particle on mica surface. A drop (2 μL) of the purified TOM complex was deposited onto an APTES‐treated mica surface and incubated for 3 min to allow partial adsorption. The surface was gently rinsed with the imaging buffer to remove unbound molecules, and then molecular dynamics were measured in a liquid cell containing ~60 μL of the same buffer. The data collection was conducted using UMEX, custom‐built software.

In the analysis of the interaction between the substrate protein pSu9‐DHFR and the TOM complex, 5 μL of substrate was added to the buffer chamber twice during observation of the TOM complex. After a few minutes, pSu9‐DHFR gradually began to appear on the mica surface, and its molecular behavior was analyzed. For tracking the interaction between the anti‐PA‐tag antibodies and the PA‐tagged TOM complex, we added one‐tenth the amount of antibody (Anti PA tag, Rat Monoclonal Antibody (FUJIFILM)) to the cross‐linked PA‐tagged TOM complex. After incubating for 30–90 min, HS‐AFM analysis was performed.

Simulated AFM images were analyzed by the BioAFMviewer (Amyot and Flechsig 2020) with a probe curvature radius of 10 nm, a cone angle of 10 degrees, and a pixel size of 1 nm.

### 4.7 Quantitative Analysis of TOM Particles by HS‐AFM

HS‐AFM images were acquired over wider scan areas, and a total of 121 TOM particles collected from multiple scan areas were analyzed using UMEX Height Analyzer. For each particle, line profiles were obtained along the long and short axes. The full width at half maximum (FWHM) and maximum height were determined from each line profile. The FWHM was defined as the width of the particle profile at 50% of its maximum height. The mean of the maximum heights obtained from the long‐ and short‐axis profiles was used as the particle height. Assuming an ellipsoidal shape, the apparent particle volume was calculated as:

$$V=\left(\pi /6\right)\times \text{FWHM}\_\text{long}\times \text{FWHM}\_\text{short}\times H$$

where FWHM_long and FWHM_short represent the FWHM values along the long and short axes, respectively, and H represents the mean particle height. The distributions of the long‐axis FWHM, short‐axis FWHM, particle height, and apparent particle volume were analyzed without prior classification of the particles as trimers, dimers, or monomers.

The 121 particles analyzed in this dataset were obtained from multiple scan areas during the same HS‐AFM measurement session using an independently purified TOM preparation and were used to characterize the particle population under a consistent imaging condition.

### 4.8 Quantitative Analysis of Substrate‐TOM Interactions by HS‐AFM

Substrate–TOM association was evaluated from line profiles of individual pSu9‐DHFR particles. The FWHM of each substrate particle was used to define its spatial extent. A substrate particle was classified as TOM‐associated when its line profile overlapped with that of a TOM particle such that the FWHM of the substrate particle could not be separately resolved. In contrast, when the FWHM of the substrate particle could be clearly resolved without overlapping with the TOM particle profile, the substrate was classified as unassociated.

To quantify substrate movement, the xy coordinates of the maximum‐height position of each substrate particle were tracked in sequential HS‐AFM frames. The frame‐to‐frame displacement from frame *n* to frame *n* + 1 was calculated from the change in xy coordinates and divided by the frame interval (0.12 s) to obtain the movement speed. Each frame‐to‐frame movement was classified as TOM‐associated or unassociated according to the substrate–TOM association state in frame *n*. Because individual substrate particles could repeatedly associate with and dissociate from TOM complexes during observation, multiple frame‐to‐frame measurements could be obtained from the same substrate particle. In total, 87 TOM‐associated and 54 unassociated frame‐to‐frame measurements were obtained from nine substrate particles.

### 4.9 Cryo‐EM Analysis and Data Processing

The TOM complex without cross‐link was concentrated up to 16.5 mg/mL and loaded onto the Chameleon system (SPT Labtech). A total of 40 nL of the sample solution was dispensed to the glow‐discharged Quantifoil Active Au 300‐mesh R1.2/2 grids with Cu nanowires (SPT Labtech) and the grids were plunged into liquid ethane and frozen in liquid nitrogen. The cross‐linked TOM complex sample was concentrated up to 10.9 mg/mL and loaded onto a glow‐discharged Quantifoil R1.2/1.3, Au,300 mesh grids. The grids were blotted by Vitrobot Mark IV (Thermo Fisher Scientific) and plunge‐frozen in liquid ethane.

Micrographs for all datasets were collected with a Titan Krios G3i microscope (Thermo Fisher Scientific) running at 300 kV and equipped with a Gatan Quantum‐LS Energy Filter (GIF) and a Gatan K3 Summit direct electron detector in the electron counting mode (The University of Tokyo, Japan). Datasets were collected with a total dose of approximately 50 electrons per Å^2^ per 48 frames by the standard mode, using the EPU software (Thermo Fisher Scientific). The dose‐fractionated movies were subjected to beam‐induced motion correction and dose weighting using Patch Motion Correction, and the contrast transfer function (CTF) parameters were estimated using Patch‐based CTF estimation in cryoSPARC v4.4.0 (Punjani et al. 2017).

Details of data collection and image processing are described in Table S5. Cryo‐EM data were processed by using cryoSPARC v4.4.0 (Punjani et al. 2017). For the TOM complex without cross‐link, 4,987,912 particles were initially picked from the 8397 motion‐corrected and dose‐weighted micrographs using Template picker, followed by several rounds of reference‐free 2D classification to curate particle sets. The particles were further curated by heterogeneous refinement, using maps derived from ab initio reconstruction as templates. The resulting 290,817 particles were subjected to non‐uniform refinement, yielding a map at overall resolutions of 3.57 Å according to the FSC criterion of 0.143 (Rosenthal and Henderson 2003; Punjani et al. 2020). Molecular graphics and cryo‐EM density map figures were prepared using CueMol (http://www.cuemol.org) and UCSF ChimeraX (Pettersen et al. 2021). The cryo‐EM density map has been deposited in the Electron Microscopy Data Bank under accession code EMD‐81859.

## Author Contributions

**Nanako Kobayashi:** conceptualization, methodology, investigation, formal analysis, funding acquisition, writing – original draft. **Satoshi N. Omura:** investigation, formal analysis, writing – original draft. **Kana Kuzasa:** investigation. **Kenichiro Imai:** investigation, formal analysis. **Shiho Kawai:** investigation. **Hirotatsu Imai:** investigation, formal analysis. **Romain Amyot:** investigation, formal analysis. **Kenichi Umeda:** investigation, formal analysis. **Osamu Nureki:** supervision. **Toshiya Endo:** conceptualization, writing – review and editing. **Noriyuki Kodera:** conceptualization, investigation, formal analysis, validation, writing – review and editing. **Yuhei Araiso:** conceptualization, formal analysis, investigation, supervision, project administration, funding acquisition, writing – original draft.

## Funding

This work was supported by JSPS KAKENHI to Y.A. (20H02583, 22K19268, and 23H01814) and in part by JSPS KAKENHI Grant Number 24KK0138, JST FOREST program (JPMJFR200F) to Y.A. and JST SPRING program (JPMJSP2135) to Na.K. Y.A. was also supported by Leading Initiative for Excellent Young Researchers, MEXT, Japan. The following grants are also acknowledged: Platform Project for Supporting Drug Discovery and Life Science Research (Basis for Supporting Innovative Drug Discovery and Life Science Research (BINDS)) from AMED under grant numbers JP25am121002 (support number 3272) (to O.N.) and JP26ama121029j0005 (support number 4859) (to K.I.); Hokuriku Bank Research Grant for Young Scientists (to Y.A.); The Mitsubishi Foundation Research Grants in the Natural Science (to Y.A.); and Naito Memorial Research Grant for Supporting the Development of the Next Generation (to Y.A.).

## Ethics Statement

The authors have nothing to report.

## Conflicts of Interest

The authors declare no conflicts of interest.

## Supporting information


**Figure S1:** (A) Purification of the TOM complex. The SDS‐PAGE gel with silver staining (left) and western blot analyses (right). The purified TOM complex contains Tom40‐Strep, Tom22‐His and Tom20 as confirmed by immunoblotting. (B) Frame‐averaged HS‐AFM image of the TOM complex obtained from sequential images. The averaged image shows a rounded morphology with a prominent central density. Imaging conditions were 20 frames per second with a scan size of 50 × 35 nm^2^ and a pixel resolution of 80 × 28. The uncropped images were included in Figure S11.
**Figure S2:** Independent HS‐AFM observations of deformation and dissociation of the TOM complex. (A, B) Two additional independent observations of TOM complex particles are shown. Raw images and corresponding annotated images are presented to facilitate interpretation of the structural changes. The trimers, deformation intermediates, dimers, monomers and antibodies are circled in blue, light gray, green, pink, and yellow, respectively. (B) The TOM complex imaged in the presence of an anti‐PA‐tag antibody is shown. Imaging conditions were either 1.43 frames per second with a scan size of 200 × 200 nm^2^ and a pixel resolution of 320 × 80 in (A), or 2.5 frames per second with a scan size of 120 × 84 nm^2^ and a pixel resolution of 400 × 70 in (B). The molecular images used in this figure were also cropped from the original data. The uncropped images were included in Figure S12. (C) Representative HS‐AFM images and line profiles used for quantitative analysis of TOM particles. Representative HS‐AFM images of TOM particles and corresponding line profiles along the long and short axes are shown. Imaging conditions were 2 frames per second with a scan size of 150 × 105 nm^2^ and a pixel resolution of 280 × 49.
**Figure S3:** Cryo‐EM analysis of the TOM complex without crosslinking. (A) Representative cryo‐EM micrograph, recorded on a 300‐kV Titan Krios microscope with a K3 camera. (B) Representative 2D class average images. (C) Final cryo‐EM 3D reconstruction of the same TOM complex sample used for HS‐AFM.
**Figure S4:** Purification of the triple‐tagged TOM complex. (A) Evaluation of the expression levels of the Tom40 fused to the Strep II, 3 × FLAG and PA ‐tags, respectively. (B) Western blot analysis for sample evaluation from independent purification of the triple‐tagged TOM complex. (C) The gel filtration chromatograms of the TOM complex (upper, navy) and the triple‐tagged TOM complex (lower, orange). The uncropped images were included in Figures S13 and S14.
**Figure S5:** Sample evaluation of the crosslinked TOM complex by using GraFix method. SDS‐PAGE analyses for the TOM complex (A) and the PA‐TOM complex (B). BN‐PAGE analyses for the TOM complex (C) and the PA‐TOM complex (D). Representative 2D class average images of the same crosslinked TOM complex sample by GraFix method used for HS‐AFM (E). The uncropped images were included in Figure S15. (F) Representative HS‐AFM image showing the TOM‐substrate interaction. The magenta dashed lines indicate the half‐height level of the substrate. On the left side of the substrate, the profile crosses the half‐height level (green dashed line), whereas on the TOM‐facing side, the local minimum (black arrow) remains above the half‐height level. Thus, FWHM could not be defined, and the substrate was therefore classified as being bound to the TOM complex.

**Figure S6:** The uncropped version of the membranes, gel and AFM images in Figure 1.
**Figure S7:** The uncropped version of the membranes AFM images in Figure 2.
**Figure S8:** The uncropped version of the membranes in Figure 3.
**Figure S9:** The uncropped version of the AFM images in Figure 4.
**Figure S10:** The uncropped version of the membranes and gel in Figure 5.
**Figure S11:** The uncropped version of the AFM images in Figure S1.
**Figure S12:** The uncropped version of the membranes in Figure S2.
**Figure S13:** The uncropped version of the membrane in Figure S4.
**Figure S14:** The uncropped version of the membranes and gels in Figure S4.
**Figure S15:** The uncropped version of the membranes and gels in Figure S5.

**Movie 1.** HS‐AFM movie showing the time‐dependent dissociation of the trimeric TOM complex into a dimer and a monomer (related to Figure 2A).

**Movie 2.** HS‐AFM movie showing the time‐dependent dissociation of the trimeric TOM complex into a dimer and a monomer during substrate addition (related to Figure 2C).

**Movie 3.** HS‐AFM movie showing the time‐dependent dissociation of the trimeric TOM complex into a dimer and a monomer (related to Figure S2A).

**Movie 4.** HS‐AFM movie showing the time‐dependent dissociation of the trimeric TOM complex into a dimer and a monomer in the presence of the anti‐PA tag antibody (related to Figure S2B).

**Movie 5.** HS‐AFM movie showing the real‐time interaction between the TOM complex and a substrate protein (related to Figure 5B).

**Movie 6.** HS‐AFM movie showing the real‐time interaction between the TOM complex and a substrate protein (related to Figure 5C).

**Table S1:** Yeast strains used in this study.
**Table S2:** Primers used in this study.
**Table S3:** Plasmids used in this study.
**Table S4:** 17‐steps gradient buffer used in this study.
**Table S5:** Data collection and processing for TOM complex.

## Data Availability

The cryo‐EM density map has been deposited in the Electron Microscopy Data Bank (www.ebi.ac.uk/pdbe/emdb/) under accession code EMD‐81859. All other data supporting the findings of this study are available from the corresponding author upon reasonable request.
