# Supplementary material for: Characterization of the Trimeric TOM Complex by HS‐AFM Single‐Molecule Analysis
Source: Genes Cells. 2026 Oct 24;31(6):e70154. doi: 10.1111/gtc.70154 (PMC13695020; doi:10.1111/gtc.70154)
Supplement: Supplementary file 1 [file GTC-31-0-s002.docx]

**Table S1 | Yeast strains used in this study.**

| Organism | Strain | Genotype | Source |
| --- | --- | --- | --- |
| *S. cerevisiae* | W303-1A | *MAT*a *leu2-3,112 trp1-1 can1-100 ura3-1 ade2-1 his3-11,15* | R. Rothstein (Columbia University, NY, NY) |
| *S. cerevisiae* | TOM All OE | *MAT*a *leu2-3,112 trp1-1 can1-100 ura3-1 ade2-1 his3-11,15, ura3-1::PGAL1-* *Tom40-GGlinker-Strep-tagⅡ-TENO1-PGAL1-Tom22-10×His TENO1-PGAL1-Tom20 TENO1-PGAL1-Tom7 TENO1-PGAL1-Tom6 TENO1-PGAL1-Tom5 TENO1-URA3* | This study |
| *S. cerevisiae* | TOM-3tag TOM All OE | *MAT*a *leu2-3,112 trp1-1 can1-100 ura3-1 ade2-1 his3-11,15, ura3-1::PGAL1-* *Tom40-GGlinker-Strep-tagⅡ-TENO1-PGAL1-Tom22-10×His TENO1-PGAL1-Tom20 TENO1-PGAL1-Tom7 TENO1-PGAL1-Tom6 TENO1-PGAL1-Tom5 TENO1-URA3, leu2-3::PGAL1-*Tom40-3×FLAG *TENO1-PGAL1-*Tom40-GGlinker-PA *TENO1-LEU2* | This study |
| *S. cerevisiae* | Tom20PA TOM All OE | *MAT*a *leu2-3,112 trp1-1 can1-100 ura3-1 ade2-1 his3-11,15, ura3-1::PGAL1-* *Tom40-GGlinker-Strep-tagⅡ-TENO1-PGAL1-Tom22-10×His TENO1-PGAL1-Tom20-PA TENO1-PGAL1-Tom7 TENO1-PGAL1-Tom6 TENO1-PGAL1-Tom5 TENO1-URA3* | This study |

**Table S2 | Primers used in this study.**

| Primer | Sequence 5′->3′ |
| --- | --- |
| Tom40 pYTK001 Fw | ATAACCGTAGTCGGCGAGACGGGTCTCATATGATGTCTGCACCAACTCCATTAG |
| Tom40 pYTK001 Rv | TTATTGGTCTGGTCAGAGACGGGTCTCAGCCATTATTTTTCGAACTGCGGGTGGCTCCATCCACCCAATTGAGGAAGAGCTTGCAATG |
| Tom22 pYTK001 Fw | ATAACCGTAGTCGGCGAGACGGGTCTCATATGATGGTCGAATTAACTGAAATTAAAG |
| Tom22 pYTK001 Rv | TTATTGGTCTGGTCAGAGACGGGTCTCAGCCATTAGTGATGATGGTGATGGTGGTGATGATGGTGTCCACCATTGGCTGTTGCTGCAGCATC |
| Tom20 pYTK001 Fw | ATAACCGTAGTCGGCGAGACGGGTCTCATATGATGTCCCAGTCGAACCCTATC |
| Tom20 pYTK001 Rv | TTATTGGTCTGGTCAGAGACGGGTCTCAGCCATCAGTCATCGATATCGTTAGCTTC |
| Tom7 pYTK001 Fw | ATAACCGTAGTCGGCGAGACGGGTCTCATATGATGAGTTTCCTACCATCTTTTATC |
| Tom7 pYTK001 Rv | TTATTGGTCTGGTCAGAGACGGGTCTCAGCCATTAAACACTTGGTAACGGAGAC |
| Tom5 pYTK001 Fw | ATAACCGTAGTCGGCGAGACGGGTCTCATATGATGTTTGGTCTACCTCAACAGG |
| Tom5 pYTK001 Rv | TTATTGGTCTGGTCAGAGACGGGTCTCAGCCATTATTTCCATTGCTTTTTCACC |
| Tom40-3×FLAG iPCR Fw | CATGACATCGACTACAAGGATGACGATGACAAGTAATGGCAGCTTTTGATTAAGCCTTC |
| Tom40-3×FLAG iPCR Rv | ATCTTTATAATCACCGTCATGGTCTTTGTAGTCCAATTGAGGAAGAGCTTGCAATGG |
| Tom40-PA iPCR Fw | GCTGAAGATGATGTTGTTTAATGGCTGAGACCCGTCTCTGAC |
| Tom40-PA iPCR Rv | ACCTGGCATAGCAACACCTCCACCCAATTGAGGAAGAGCTTG |
| Tom20-PA iPCR Fw | GCTGAAGATGATGTTGTTTGATGGCAGCTTTTGATTAAGCCTTC |
| Tom20-PA iPCR Rv | ACCTGGCATAGCAACACCGTCATCGATATCGTTAGCTTCAGCAAC |

**Table S3 | Plasmids used in this study.**

| Plasmid | Promoter | Expressed protein | Source |
| --- | --- | --- | --- |
| pYTK096/TOM complex | *GALⅠ* | Tom40-GGlinker-Strep-tagⅡ  Tom22-10×His  Tom20  Tom7  Tom6  Tom5 | This study |
| pYTK096(LEU2ver.)/TOM complex-Tom40-2tags | *GALⅠ* | Tom40-3×FLAG  Tom40-GGlinker-PA | This study |
| pYTK096/TOM complex-Tom20PA | *GALⅠ* | Tom40-GG-Strep-tagⅡ  Tom22-10×His  Tom20-PA  Tom7  Tom6  Tom5 | This study |
| pET22b/pSu9-mDHFR | *T7* | pSu9-mouseDHFR | T. Endo (Kyoto Sangyo University, Kyoto, Japan) |

**Table S4 | 17-steps gradient buffer used in this study.**

| No. | Sucrose(%) | Glutaraldehyde(%) | HEPES pH7.4(mM) | NaCl(mM) | GDN(%) |
| --- | --- | --- | --- | --- | --- |
| 1 | 45 | 0.8 | 20 | 150 | 0.01 |
| 2 | 42.5 | 0.75 | 20 | 150 | 0.01 |
| 3 | 40 | 0.7 | 20 | 150 | 0.01 |
| 4 | 37.5 | 0.65 | 20 | 150 | 0.01 |
| 5 | 35 | 0.6 | 20 | 150 | 0.01 |
| 6 | 32.5 | 0.55 | 20 | 150 | 0.01 |
| 7 | 30 | 0.5 | 20 | 150 | 0.01 |
| 8 | 27.5 | 0.45 | 20 | 150 | 0.01 |
| 9 | 25 | 0.4 | 20 | 150 | 0.01 |
| 10 | 22.5 | 0.35 | 20 | 150 | 0.01 |
| 11 | 20 | 0.3 | 20 | 150 | 0.01 |
| 12 | 17.5 | 0.25 | 20 | 150 | 0.01 |
| 13 | 15 | 0.2 | 20 | 150 | 0.01 |
| 14 | 12.5 | 0.15 | 20 | 150 | 0.01 |
| 15 | 10 | 0.1 | 20 | 150 | 0.01 |
| 16 | 7.5 | 0.05 | 20 | 150 | 0.01 |
| 17 | 5 | 0 | 20 | 150 | 0.01 |

**Table S5 | Data collection and processing for TOM complex.**

| **Sample** | **TOM complex without cross-link** | **Cross-linked TOM complex** |
| --- | --- | --- |
| **Microscope** | Titan Krios G3i | |
| **Detector** | Gatan K3 Camera | |
| **Magnification** | 105,000 | |
| **Voltage (kV)** | 300 | |
| **Electron exposure (e^–^/Å^2^)** | 50 | |
| **Defocus range (μm)** | −0.8 to −1.6 | |
| **Pixel size (Å)** | 0.83 | |
| **Symmetry imposed** | *C_1_* | |
| **Number of movies** | 8,397 | 4,189 |
| **Initial particle images (no.)** | 4,987,912 | - |
| **Final particle images (no.)** | 290,817 | - |
| **Map resolution (Å)** | 3.57 | - |
| **FSC threshold** | 0.143 | |
